# Convolutional neural network for human cancer types prediction by integrating protein interaction networks and omics data

**DOI:** 10.1038/s41598-021-98814-y

**Published:** 2021-10-19

**Authors:** Yi-Hsuan Chuang, Sing-Han Huang, Tzu-Mao Hung, Xiang-Yu Lin, Jung-Yu Lee, Wen-Sen Lai, Jinn-Moon Yang

**Affiliations:** 1 Institute of Bioinformatics and Systems Biology, National Chiao Tung University, Hsinchu, 300 Taiwan; 2 Department of Otolaryngology–Head and Neck Surgery, Taichung Armed Forces General Hospital, Taichung, Taiwan; 3 Department of Otolaryngology–Head and Neck Surgery, Tri-Service General Hospital, National Defense Medical Center, Taipei, Taiwan

**Keywords:** Biological techniques, Cancer, Computational biology and bioinformatics

## Abstract

Many studies have proven the power of gene expression profile in cancer identification, however, the explosive growth of genomics data increasing needs of tools for cancer diagnosis and prognosis in high accuracy and short times. Here, we collected 6136 human samples from 11 cancer types, and integrated their gene expression profiles and protein–protein interaction (PPI) network to generate 2D images with spectral clustering method. To predict normal samples and 11 cancer tumor types, the images of these 6136 human cancer network were separated into training and validation dataset to develop convolutional neural network (CNN). Our model showed 97.4% and 95.4% accuracies in identification of normal versus tumors and 11 cancer types, respectively. We also provided the results that tumors located in neighboring tissues or in the same cell types, would induce machine make error classification due to the similar gene expression profiles. Furthermore, we observed some patients may exhibit better prognosis if their tumors often misjudged into normal samples. As far as we know, we are the first to generate thousands of cancer networks to predict and classify multiple cancer types with CNN architecture. We believe that our model not only can be applied to cancer diagnosis and prognosis, but also promote the discovery of multiple cancer biomarkers.

## Introduction

Cancer is the second leading cause of death with more than 9.5 million patients yearly worldwide^1^. The high mortality rate is due to late-stage diagnosis and tumor heterogeneity, which hampers the optimal decision of patient care and treatment^2,3^. Some studies have indicated that cancer diagnosis, therapy strategies and prognosis evaluation relied on medical images, such as histopathology image, computed tomography (CT), tumor nodal metastasis and tumor extranodal extension. These images were identified and classified subjectively by inter-observer, whereas the agreements are moderate and disturbing (kappa coefficient = 0.4–0.7)^4–6^. To provide suitable treatment and increase survival for patients, recent researches started to apply deep learning techniques on biomedical applications. For example, the convolutional neural network (CNN) techniques were used to identify the metastasis indicators, cancer cell types and molecular subtypes by using medical images and omics data, providing critical information for next therapeutic management^4,7–11^.

As the increasing biological data were released and available, many studies have exhibited the powers of omics data in discovering biomarkers and classifications for cancers. Some studies indicated that different cancer types would regulate distinct genes and pathways, which might affect treatment efficacy and clinical outcome^12,13^. Several machine learning (ML) and deep learning (DL) approaches were utilized to determine cancer types by using microRNA expression data of 4046 samples from 12 cancer types. They showed the promising results that ML methods were able to identify the diversity of pan-cancer by microRNA profiles^14^. Junyi et al. developed a self-normalizing neural network (SNN) and utilized Monte Carlo Feature Selection (MCFS) to distinguish four cancers by using DNA copy number variant (CNV) of 2084 patients, and obtained the higher accuracy than the ones of random forest classifier^15^. Milad et al. implemented three CNN models and collected RNA-Seq gene expression profiles from > 10,000 samples of 33 cancer types for training the models^10^. Their results showed the accuracies (94–95%) for classification of 33 cancer types and normal samples. They also discovered the tissue origin would affect the cancer type prediction, and provided the solutions to reduce the influences by combining tumor and normal samples. Furthermore, the computational models utilizing protein–protein interactions (PPIs) would predict specific biological functions in different cancer types^16^. Recently, Teppei et al. combined two kinds of biological data, the gene expression profile and human PPI network, to generate 2D representation as the input of the spectral-CNN model^17^. They obtained the prediction accuracy (81%) for classification of lung tumors and normal samples.

In this study, we aimed to develop a CNN model to identify and classify normal tissues and tumor samples of multiple cancer types. Primarily, we integrated PPI network and gene expression profile of 11 cancer types, to generate 6136 network images in 2D representation by using spectral clustering (i.e., Laplacian matrix). Where 1228 network images were used for training and testing in CNN model; and the other 4908 images, gene expression clustering and survival data were used for validation. Our results indicate that our CNN model has high accuracies (97.4% and 95.4%) for identification and classification of normal tissues and 11 cancer tumors.

## Materials and methods

**Figure 1:**
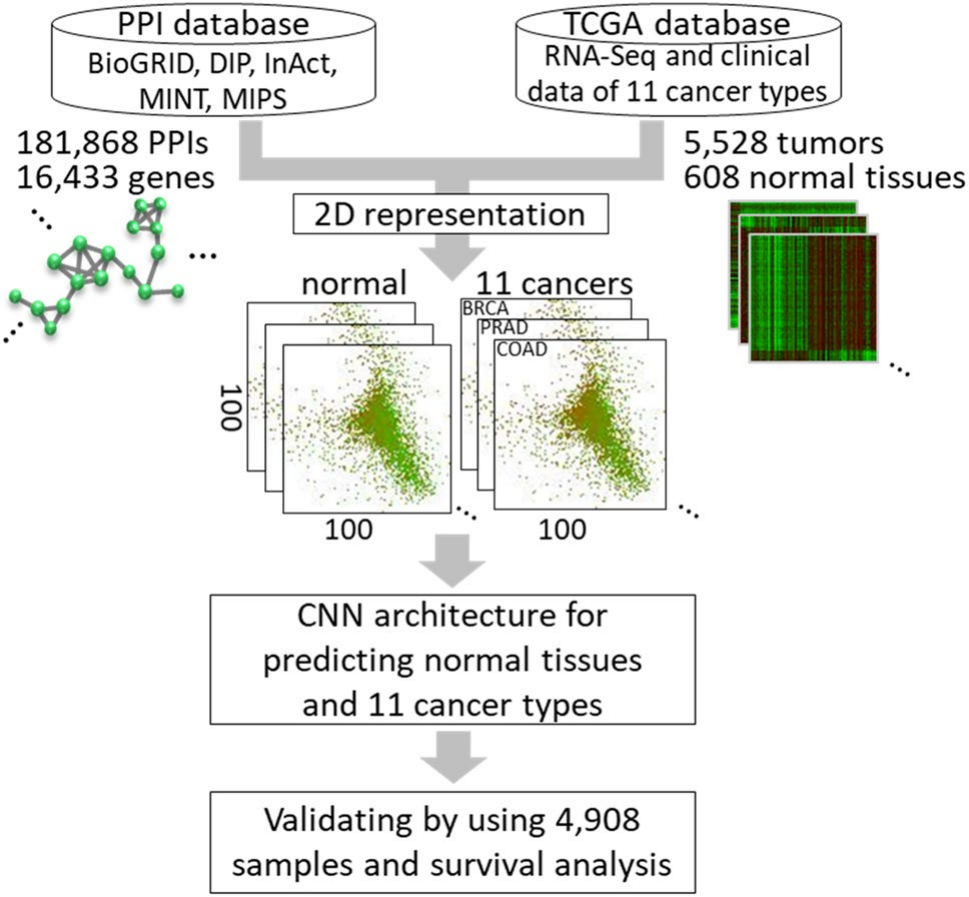
Schematic of integrating protein interaction networks and genomic profiling into convolutional neural network for multi-cancer classification. RNA-Seq data and clinical data of 6136 samples with 11 cancer types are collected from The Cancer Genome Atlas (TCGA) database, and 181,868 protein–protein interactions (PPIs) of 16,433 human proteins from five public databases. Then, Laplacian approach was utilized to map PPI network into 2D space and combined with the gene expression, to generate 608 normal and 5528 tumor sample images for convolutional neural network (CNN) model and validation dataset.

The overview of our method for predicting and classifying normal tissues and tumors of 11 cancer types is presented in Fig. 1. First, we collected PPIs and clinical gene expression data, and generate 6136 network images in 2D space by using spectral clustering. Then, a CNN model was constructed and used to discriminate normal tissues, tumors and cancer types from the network images. The accuracy (ACC) was calculated to determine the powers for prediction and classification for 11 cancer types. Finally, the confusion matrix and survival data were performed to explain model abilities. (The source codes and data sets were uploaded on the Github, https://github.com/bioxgem/CNN_model.git).

### Datasets

To study cancer classification, we collected level 3 RNA-Seq data and clinical data of 11 cancer types from The Cancer Genome Atlas (TCGA)^18,19^. The number samples are more than 30 tumor and 30 normal samples for each cancer type (Table 1). In total, the gene expression profiles of 20,531 genes from 5528 tumors and 608 normal tissues were collected for identification of cancer network signatures.

**Table 1:** Expression datasets of RNA-seq and clinical data in 11 cancers.

Cancer abbreviation	Cancer type	No. of total normal sample	No. of total tumor sample
BRCA	Breast invasive carcinoma	113	1095
COAD	Colon adenocarcinoma	41	285
HNSC	Head and neck squamous cell carcinoma	44	520
KIRC	Kidney renal clear cell carcinoma	72	533
KIRP	Kidney renal papillary cell carcinoma	32	290
LIHC	Liver hepatocellular carcinoma	50	371
LUAD	Lung adenocarcinoma	59	515
LUSC	Lung squamous cell carcinoma	51	502
PRAD	Prostate adenocarcinoma	52	497
STAD	Stomach adenocarcinoma	35	415
THCA	Thyroid carcinoma	59	505

The human PPIs were collected from five public databases (i.e., BioGRID^20^, DIP^21^, IntAct^22^, MINT^23^ and MIPS^24^), including 16,433 human proteins and 181,868 PPIs. To combine RNA-Seq and PPIs data, we assigned proteins with gene expression using gene name and gene ID, and finally acquired 14,230 proteins and 152,519 PPIs for further analysis.

### Identification of differentially expressed genes and corresponding PPI network

We first identified differentially expressed genes (DEGs) between tumors and corresponding normal tissues for 11 cancer types by computing gene expression fold change and modified t-statistic (limma package v.3.38.3). Finally, 12,024 genes were considered as DEGs with |fold change| ≥ 2 and adjust *p* value < 0.01 in at least one cancer type. By these DEGs, we selected a maximum-subnetwork with 6261 DEGs with 28,439 PPIs and combined the gene expression profiles of 5528 tumors and 608 normal tissues. These cancer networks will be processed with dimensionality reduction utilizing spectral clustering, for cancer prediction and classification in CNN model.

### Spectral clustering and 2D representation for cancer-related network

CNN techniques have widely used to recognize medical images. However, cancer networks with interactions, nodes (proteins) and gene expression perturbation (heatmap) are more complex than images. Therefore, we used a spectral clustering approach, Laplacian (L) matrix to reduce dimensionality of complex cancer networks and applied on CNN techniques. The cancer network can be transformed into the adjacency (A) and diagonal (D) matrices to gain Laplacian (L) matrix as follows^17,25,26^:

1 $$L=D-A$$

**Figure 2:**
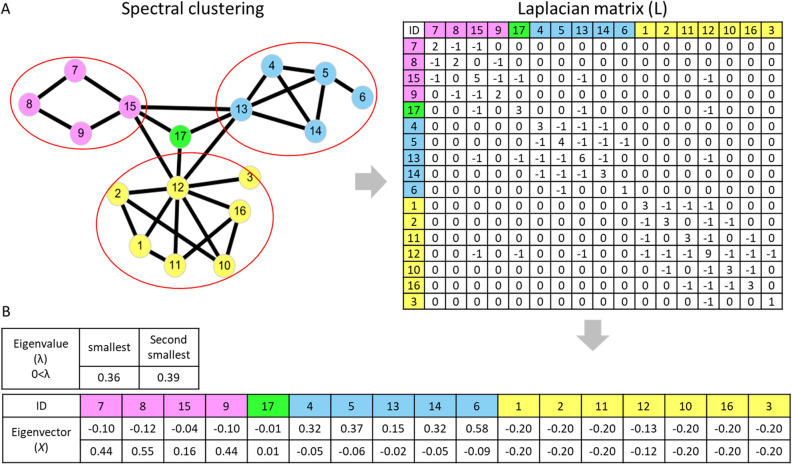
The Laplacian matrix of cancer network and 2D space representation. (**A**) The network with 17 nodes with the interaction information and its Laplacian matrix (adjacency and diagonal matrix). The value of a diagonal cell is the degree of this node, whereas “− 1” indicates the interaction is existing between two nodes, such as cells between node 7 to node 8 and 15 were assigned value “− 1” in first row; otherwise, the cells are assigned value “0” if no interaction between two nodes. (**B**) The smallest and second smallest non-negative and non-zero eigenvalues with their corresponding eigenvectors were used as x–y axis to map PPI network to 2D space.

For example, in symmetric Laplacian (L) matrix (Fig. 2A), the matrix cells were assigned value “− 1” when the two proteins had interaction, otherwise the cells were assigned value “0”; whereas the cells in diagonal were assigned value of node degree (the number of edges connected to the node in the network). Next, we obtained the eigenvalue and eigenvector of Laplacian matrix using linear transformation. To retain the network topology and connectivity, we utilized the smallest and second smallest non-negative and non-zero eigenvalues with their corresponding eigenvectors to map the cancer network (6261 DEGs and 28,439 interactions) into 2D spaces with 100 × 100 cells (Fig. 2B)^27,28^. After the dimensionality reduction for PPI network, the 1849 unique nodes were displayed in 2D representation and assigned with gene expression value of clinical samples (if numerous genes overlapped into a single node, then their gene expression was averaged and assigned to the node). In total, we generated 6136 images of cancer networks for CNN model to predict tumors, normal tissues and cancer types.

### Convolutional neural network

**Figure 3:**
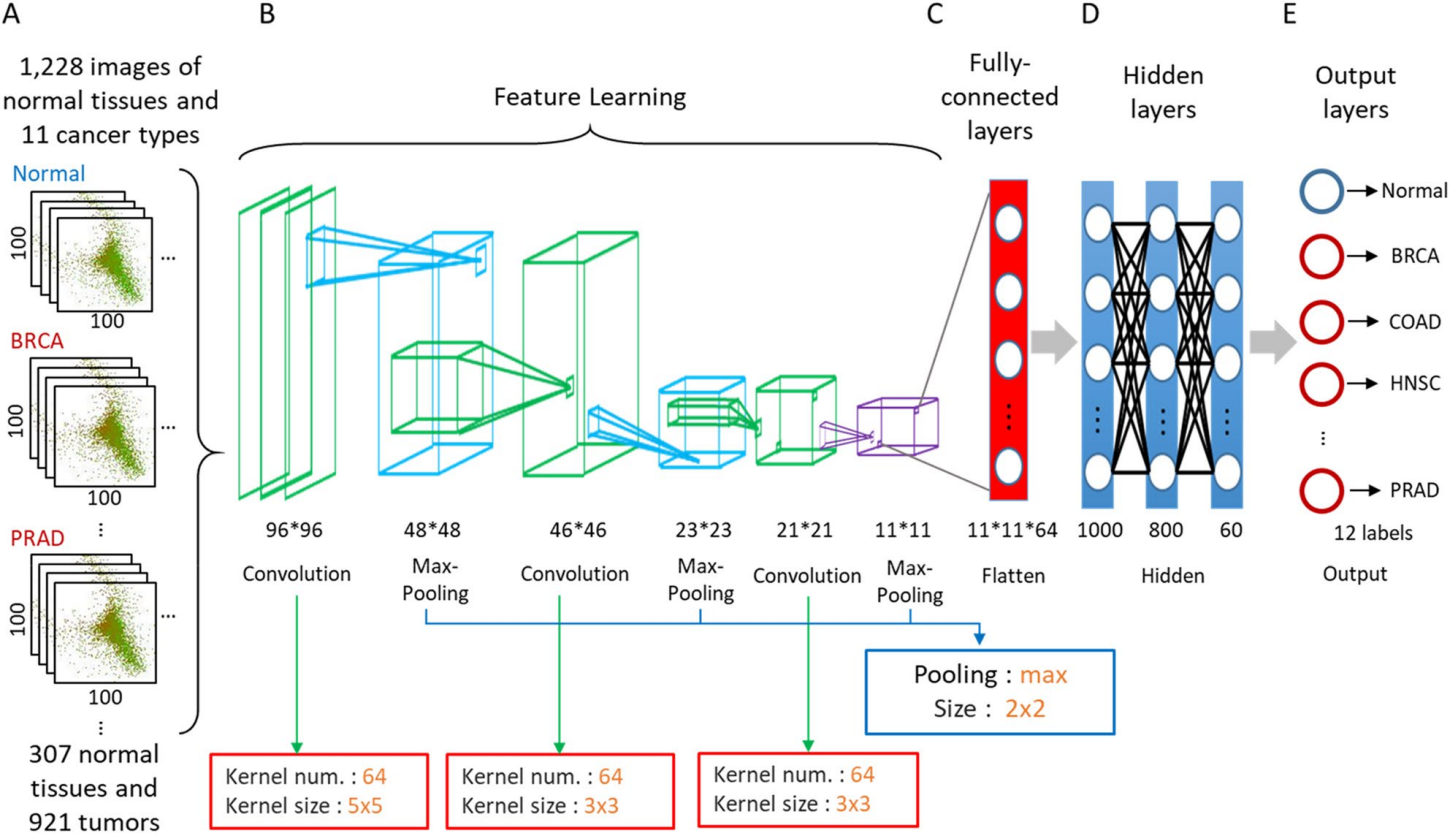
The CNN architecture of multiple cancer classification. (**A**) 1228 2D representation images with 100 × 100 cells were used as input data. Every image was processed with (**B**) three successive convolutional layers and pooling layers for feature learning. The obtained feature maps were (**C**) flatten to (**D**) train and (**E**) predict in fully-connected layers, hidden layers and output layers for normal tissues, tumors and 11 cancer types.

Our CNN architecture is displayed in Fig. 3. We first selected 1228 2D images including 307 normal tissues and 921 tumors from 11 cancer types (Fig. 3A). These 1228 images with 100 × 100 cells were separated into 75% training and 25% testing datasets, and processed with three successive convolutional layers (64 kernel matrices with sizes of 5 × 5, 3 × 3 and 3 × 3) and pooling layers (max-pooling and size of 2 × 2). In total, 64 feature maps of size 11 × 11 were extracted (Fig. 3B), and would be flattened in fully-connected layers (size of 11 × 11 × 64) and calculated in the next hidden layers (Fig. 3C). Our CNN architecture included three hidden layers with 1000, 800, and 60 neurons with rectified linear unit (ReLU) activation function (Fig. 3D). Finally, the 12 predicting results (i.e., normal tissues and 11 cancer types) were displayed in output layers (Fig. 3E).

We utilized accuracy as an indicator for training and validating the CNN models, the accuracy is defined as:

2 $$Accuracy=\frac{TP+TN}{TP+TN+FP+FN}$$

where *TP* is true positive, *TN* is true negative, *FP* is false positive and *FN* is false negative.

## Results

### Cancer-related networks

To generate cancer-related network, we first collected gene expression data of 608 normal tissues and 5528 tumors of 11 cancer types from TCGA database (Table 1). Next, we acquired a universal PPI network with 6261 genes with 28,439 interactions, and mapped this network into 2D space by utilizing the spectral clustering approach. After combining the gene expression profiles, the 6136 individual images of cancer-related networks of 11 cancer types were generated and used as training, testing and validation datasets in CNN models.

### Prediction and classification of normal tissues and tumors of 11 cancer types

**Figure 4:**
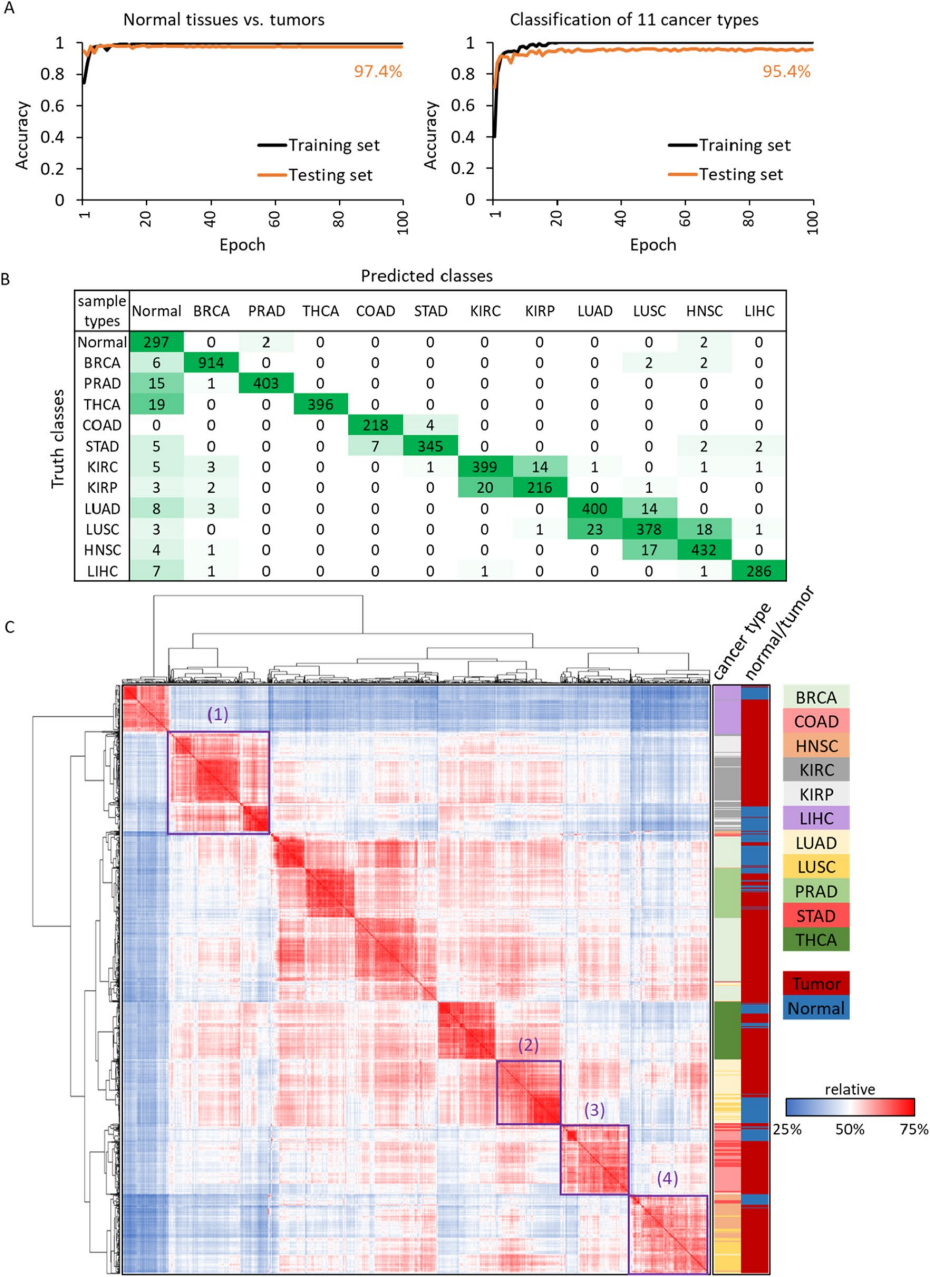
The prediction results of tumors/normal samples and confusion matrix for 11 cancers. The prediction accuracies of (**A**) normal tissues and tumors (left), as well as normal tissues and 11 cancer types (right). The x-axis is 100 training/testing times (epochs); and the y-axis presents accuracy of training/testing. (**B**) The confusion matrix is generated from 4908 independent images. The y-axis is true classes (ground truth) of validation samples and x-axis is prediction classes of CNN model. The diagonal cells are the sample counts of correct prediction/classification. (**C**) The hierarchical clustering of 1228 samples by using Pearson’s *r* of gene expression.

In total 6136 images of cancer-related networks, we first randomly selected 307 normal tissue images and 921 tumor images (25% and 75%, respectively). For example, 57 of 608 normal tissues were provided from breast invasive carcinoma (BRCA), thus we picked the amount of 171 BRCA tumors in random by using Python function “random.shuffle”; and so on, we obtained the corresponding sample size of tumors for each cancer type. Of the 1228 images, 921 (75%) tumor images and 307 (25%) normal images were used as training sets, whereas the remain 4908 sample images were used as validation in CNN model. After training 100 epochs with a stable prediction result (Fig. 4A), the training and validation (independent 4908 samples) accuracies were 97.4% and 95.4% for identification of normal tissues versus tumors, and the ones were 95.4% and 95.1% for classification of normal tissues versus 11 cancer tumors.

The confusion matrix of 4908 validation images was provided in Fig. 4B, which showed 4684 correct classifications (in diagonal cells) and 224 erroneous identifications (non-diagonal cells). For example, 914 BRCA tumors were correctly identified, and 6 and 4 tumors were classified incorrectly into normal tissues and other cancer tumors, respectively. We also discovered some interesting results that the different cancer tumors between neighboring tissues or tumors with the same cell types, were more likely to make incorrect classifications for each other. For instance, in all 224 erroneous identifications (non-diagonal cells), the misjudgments were 56% for LUAD tumors classified into LUSC. For LUSC, the misjudgments were classified into LUAD (50%) and HNSC (39.1%). The similar results were observed for kidney renal clear cell carcinoma (KIRC) and kidney renal papillary cell carcinoma (KIRP).

We use Python SciPy package^29^ (i.e., scipy.cluster.hierarchy) to cluster the gene expression profiling of normal tissues and 11 cancer tumors (Fig. 4C), and proposed several observations for the incorrect classifications for our CNN model. The tumors of neighboring tissues (i.e., KIRC vs. KIRP and LUSC vs. LUAD of purple boxes (1) and (2)) and the same cell types (i.e., adenocarcinoma, purples boxes (3); and (4)) would be clustered together and display similar gene expression profiles. These tumors with tissue-specific and cell-type-specific often generated similar network images to confuse the CNN model. We also generated the clustering of gene expression using 4908 validation samples and obtained the similar results (Supplementary Figure S1). These observations were critical and supported some studies’ conclusions for difficulties of clinical diagnosis, prognosis and treatment between LUSC to LUAD and HNSC^30–33^. In summary, our CNN model displayed the 89–99% precision for classification of 11 cancer tumors, which exhibited the potentials in medical applications.

### The random validation sets for verification of model performance

To verify our findings and CNN model, we randomly generated 50 training sets and validation sets from 6136 samples. Every training sets contained 307 normal samples and 921 tumor samples (sample size 25% and 75%, respectively), and the remaining 301 normal samples and 4607 tumor samples were considered as validation sets. there were 944 tumor samples misjudged into error cancer types, and calculated the frequency of error identification in 50 repeated experiments. For example, there was a lung adenocarcinomas (LUAD) patient, TCGA-44-5643, who was selected as validation sample 40 times, but always misjudged into lung squamous cell carcinomas (LUSC), then its frequency of error identification was 100% (40/40). The accuracy values of 50 distinct repeated experiments were displayed in Figure S2. The prediction accuracy of median, minimum, maximum and variance for 50 times repeated experiments are 95.55%, 94.8%, 96.2% and 1.05E−05, respectively. We believe our CNN model is stable and able to identify normal and 11 cancer tumor samples. The detailed accuracy value and confusion matrix of 50 repeated experiments were reported in the Supplementary File 1.

### Frequency of error identification is associated with the similarity of gene expression between cancer types

**Figure 5:**
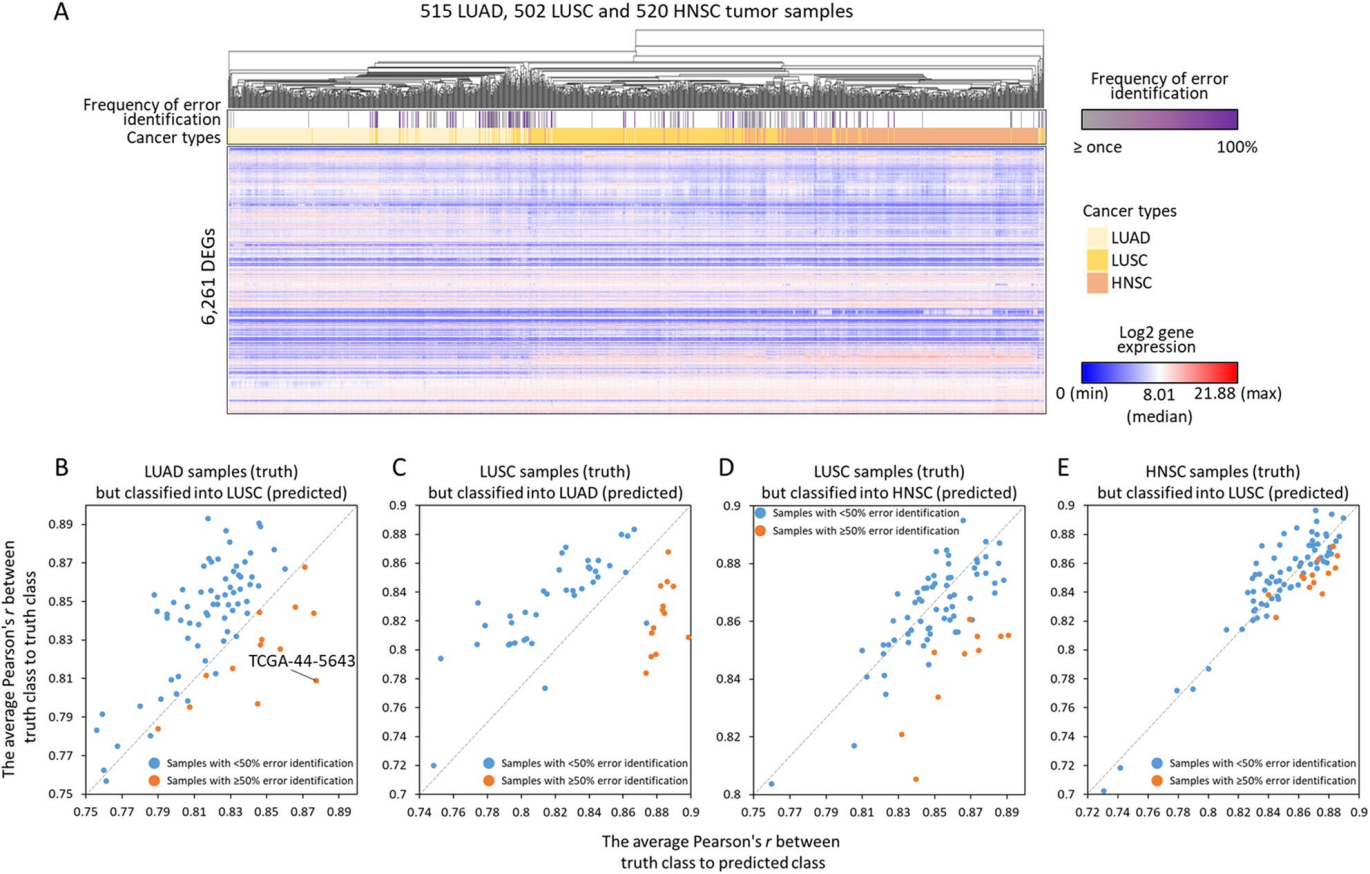
The correlation analysis of gene expression profiles between misjudged tumors and tumors in truth and predicted classes. (**A**) The heat map showed the unsupervised clustering analysis of gene expression in LUAD, LUSC and HNSC tumor samples. The intensity of gene expression for 6261 DEGs are presented in red color and blue color. To the top panels, three colors indicate LUAD (light yellow), LUSC (gold color) and HNSC (peach color) respectively. And the frequency of error identification is presented in grey (≥ 1 misjudgment) and purple color. In the four scatter plots (**B**–**E**), each dot represents the each misjudged tumor sample, and the orange dots means their frequencies of error identification ≥ 50% among the number of total prediction times, otherwise the ones were presented with blue dots. The plots illustrated the averaged gene expression correlation (i.e., Pearson’s *r*) between the each misjudged tumor of (**B**) LUAD, (**C**, **D**) LUSC and (**E**) HNSC to every tumor in truth class cancers and in predicted class cancers. If the dots are below the diagonal that indicate the gene expression profiles of misjudged tumors are more similar to tumors in predicted classes than in truth classes.

Based on the prediction results of 11 cancers (Fig. 4B,C), the tissue-specific and cell-type-specific may influence CNN model on identification of 11 cancer tumor samples. To confirm the assumptions, we performed the hierarchical clustering of gene expression in 515 lung adenocarcinomas (LUAD), 502 lung squamous cell carcinomas (LUSC) and 520 head and neck squamous cell carcinomas (HNSC) by using Pearson's *r*, to study the gene expression correlations of distinct cancer tumors in adjacent tissues and same cell-types. The clustering was generated by online tool, Morpheus (https://software.broadinstitute.org/morpheus) and displayed in Fig. 5A, the tumor samples in the same cancer type would be clustered together generally; however, in the 50 times repeated experiments, the misjudged samples which obtained higher frequencies of error identification (purple color) were often interspersed within other cancer type, that because of their gene expression profile were considered more similar to tumors of predicted classes than truth classes.

The scatter plots (Fig. 5B–E) exhibited the gene expression correlations between each of misjudged sample to every tumor in truth class cancers and to every tumor in predicted class cancers. There was a LUAD sample of patient TCGA-44-5643, for instance, was appeared 40 times in 50 random sets, but always be identified into LUSC incorrectly (frequency of error identification = 100%; Fig. 5B); we then used the gene expression profile of 6261 DEGs to calculate and average the correlations (i.e., Pearson's *r*) between this sample (i.e., LUAD tumor of TCGA-44-5643) to every LUAD samples and to every LUSC samples. In this example, the means of correlations to LUAD and LUSC were 0.809 and 0.877, respectively, that indicated the gene expression profile of this LUAD sample was more similar to LUSC tumors than LUAD tumors. The same conclusions were observed in the LUSC and HNSC cancer types, because the misjudged tumors presented higher similarity of gene expression profiles to predicted class cancers compared to the truth class cancers. (Fig. 5C–E), and this situation would occur more frequently for the samples which contained ≥ 50% error identification (orange dots). Additional analysis of misjudged tumors in KIRC and KIRP, and COAD and STAD also arrived same results (Figs. S3 and S4).

### The association of misjudgments between tumors and normal samples and survival time

Another interesting result is that, of all misjudgments in the 50 repeated experiments, the thyroid carcinoma (THCA), prostate adenocarcinoma (PRAD) and liver hepatocellular carcinoma (LIHC), and breast invasive carcinoma (BRCA) displayed 54 to 100% misjudgments of identifying tumors into normal samples (Table 2). And in these four cancer types, for the dead patients, the tumors misjudged into normal showed better prognosis (lived ≥ 2 years) compared with the ones identified correctly. For example, in the THCA cancer, there were 37 tumors misjudged into normal samples, 468 tumors were correctly identified, and 3 and 6 samples were dead (but lived ≥ 2 years) in the misjudged samples and correctly identified samples, respectively; the odds ratio of two groups (i.e., error and correct identification samples) in survival time was 6.324 that indicated the tumors misjudged into normal samples were exhibited longer survival time or less number of death in THCA cancer. According to the previous results (Fig. 5), the patients who were misjudged into normal tissues might display more similar gene expression profiles as normal tissues, and had better prognosis.

**Table 2:** The association between the number of misjudgments between tumors and normal samples and survival time.

Cancer types	Frequency of tumor misjudged into normal (%)	No. of tumors with error identifications	No. of tumors misjudged into normal	No. of correctly identified samples	The misjudged samples which were dead, but alive ≥ 2 years	The correctly identified samples which were dead, but alive ≥ 2 years	Odds ratio
THCA	100.00	37	37	468	3	6	6.324
PRAD	96.61	59	57	438	No death	4	NA
LIHC	70.83	24	17	347	4	19	4.297
BRCA	54.84	31	17	1064	2	64	1.956
STAD	38.46	91	35	324	2	19	0.974
KIRC	29.09	55	16	478	1	60	0.498
LUAD	22.55	102	23	413	1	44	0.408
HNSC	15.53	103	16	417	0	39	0
KIRP	15.15	66	10	224	0	10	0
LUSC	6.90	145	10	357	1	50	0.714
COAD	4.17	24	1	261	No clinical data	12	NA

*NA* not available.

## Discussions

The related study developed three CNN models and discovered that the same tissue of origin, such as lung adenocarcinoma (LUAD) and lung squamous cell carcinoma (LUSC), led the model to make misjudgments^10^. In our study, we obtained the similar observations, and further observed that two different cancer types, LUSC and HNSC with the same cell type (both were squamous cell) would also confuse CNN model (Fig. 4C). Furthermore, we indicated that the LUSC tumor samples, which were often misjudged into HNSC, showed more similar gene expression profiles to HNSC, and vice versa. We consider our works have sensitivity in recognizing cell-type-specific, and displayed potential on prediction of prognosis (e.g., metastasis) and treatment selection for LUAD, LUSC and HNSC in the future^34–36^.

We also compared our CNN model with the ones of Mostavi et al.^10^ and Matsubara et al.^17^ (Supplementary Table S1). First, the CNN model of Matsubara et al. is similar to the 2D-Vanilla-CNN of Mostavi et al., but has two more convolution and pooling layers. Our CNN model was similar to Matsubara et al.; however, we modified CNN structure to predict multi-cancer types. Second, our model and Mostavi et al. filtered the overexpression genes before reformatting the data to model input, but Matsubara et al. did not. Third, we and Matsubara et al. integrated gene expression profiles and PPI networks as model inputs. Fourth, we and Mostavi et al. performed multi-label classification, but Matsubara et al. did not. In summary, our CNN model has several advantages, such as the multi-label classification, DEGs filtering and integration of multi-omics data, and the better accuracies.

In general, our model is able to integrate multi-omics data with protein–protein interactions (PPIs) for the classification of different cancer types if the omics data can be presented in numerical values and mapped into PPI networks. However, our CNN model has several limitations. First, except the RNA-Seq gene expression profile, many researches provided the evidences of multiple omics data (e.g., alternative polyadenylation, microbiota or antigen) for identifying cancer types^37–39^, but some biological data were not easy to map into PPI networks for generating our model input data. Second, after implementing dimensionality reduction for PPI network by spectral clustering approach, the topology of PPI networks would be changed and some nodes (i.e., proteins) of the network were overlapped into new ones; that led us to trace proteins back difficultly, and made the results cannot be interpretable. However, we provide the promising results for identification and classification of pan-cancer by integrating gene expression and PPI networks.

## Conclusions

In this study, we are the first to combine multi-cancer gene expression profiles and PPI networks for CNN architecture to identify and classify thousands of normal tissues and tumors. Our CNN model provided 95.4% accuracy of classification between normal tissues and 11 cancer types. Furthermore, we provided some evidences about the different cancer types that would display similar gene expression signature in biological networks due to tissue-specific and cell-type-specific, that confuse machine to identify the truths.

## Supplementary Information

Supplementary Information 1.

Supplementary Information 2.

Supplementary Information 3.
