# Supplementary material for: Convolutional neural network for human cancer types prediction by integrating protein interaction networks and omics data
Source: Sci Rep. 2021 Oct 19;11:20691. doi: 10.1038/s41598-021-98814-y (PMC8526703; doi:10.1038/s41598-021-98814-y)
Supplement: Supplementary file 2 — Supplementary Information 2. [file 41598_2021_98814_MOESM2_ESM.docx]

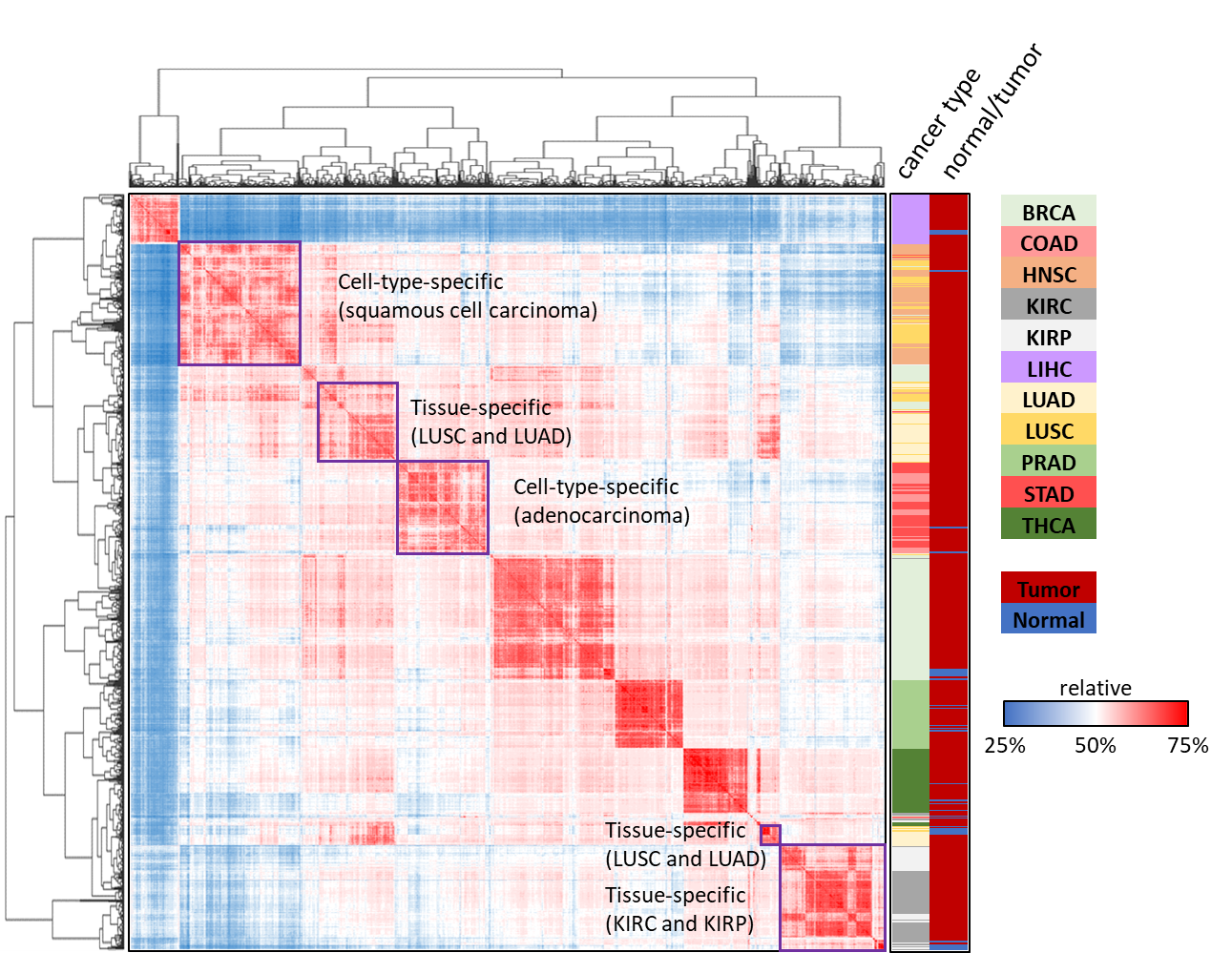


**Figure S1.** The hierarchical clustering of 4,908 validation samples by using Pearson’s *r* of gene expression. The results of gene expression clustering displayed cell-type-specific (i.e., squamous cell carcinoma and adenocarcinoma) and tissue-specific (i.e., LUSC vs. LUAD and KIRC vs. KIRP), which suggested that the similar gene expression profiles might confuse CNN model.


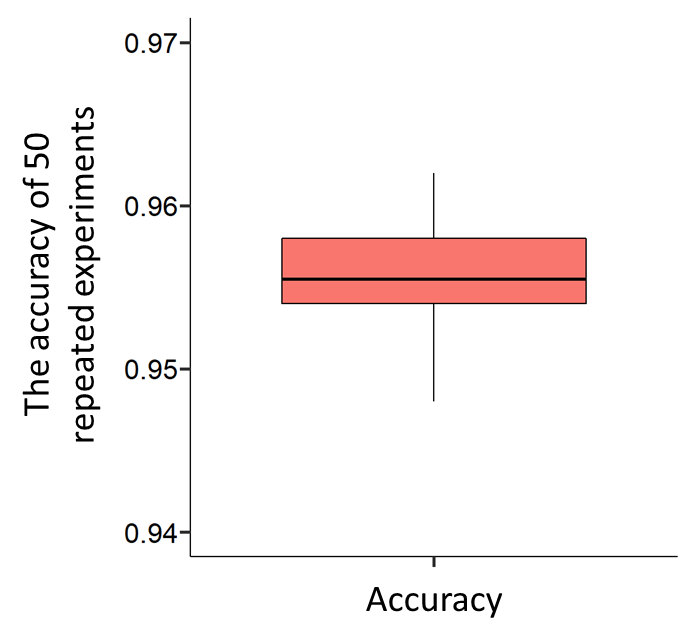


**Figure S2.** The accuracy values of 50 distinct repeated experiments.


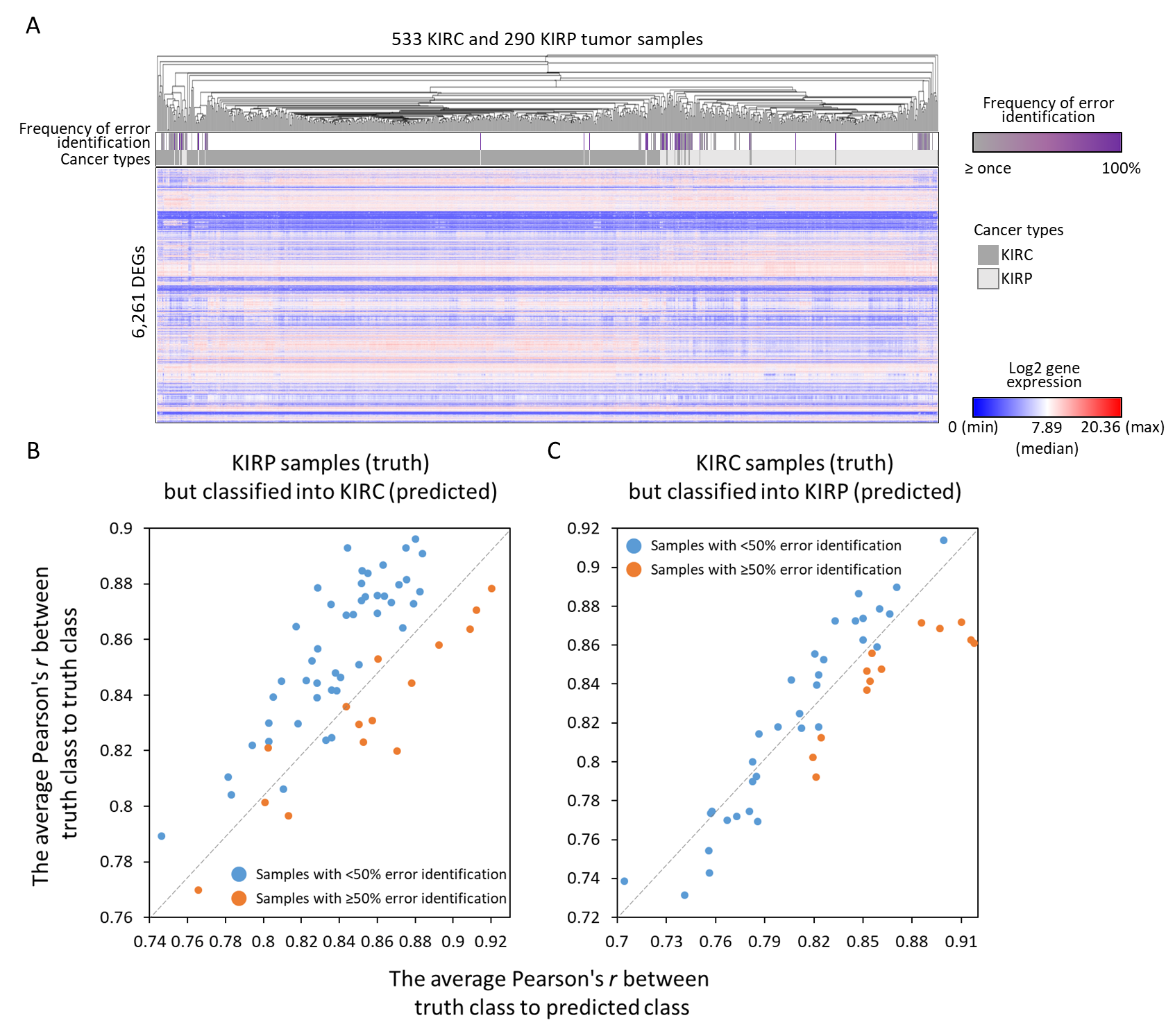


**Figure S3.** The correlation analysis of gene expression profiles between misjudged tumors and tumors in truth and predicted classes. (A) The heat map showed the unsupervised clustering analysis of gene expression in KIRC and KIRP tumor samples. The intensity of gene expression for 6,261 DEGs are presented in red color and blue color. To the top panels, two colors indicate KIRC (grey color) and KIRP (light grey), respectively. And the frequency of error identification is presented in purple color. In the two scatter plots (B and C), each dot represents the each misjudged tumor sample, and the orange dots means their frequencies of error identification ≥ 50% among the number of total prediction times, otherwise the ones were presented with blue dots. The plots illustrated the averaged gene expression correlation (i.e., Pearson’s *r*) between the each misjudged tumor of (B) KIRP and (C) KIRC to every tumor in truth class cancers and in predicted class cancers. If the dots are below the diagonal that indicate the gene expression profiles of misjudged tumors are more similar to tumors in predicted classes than in truth classes.


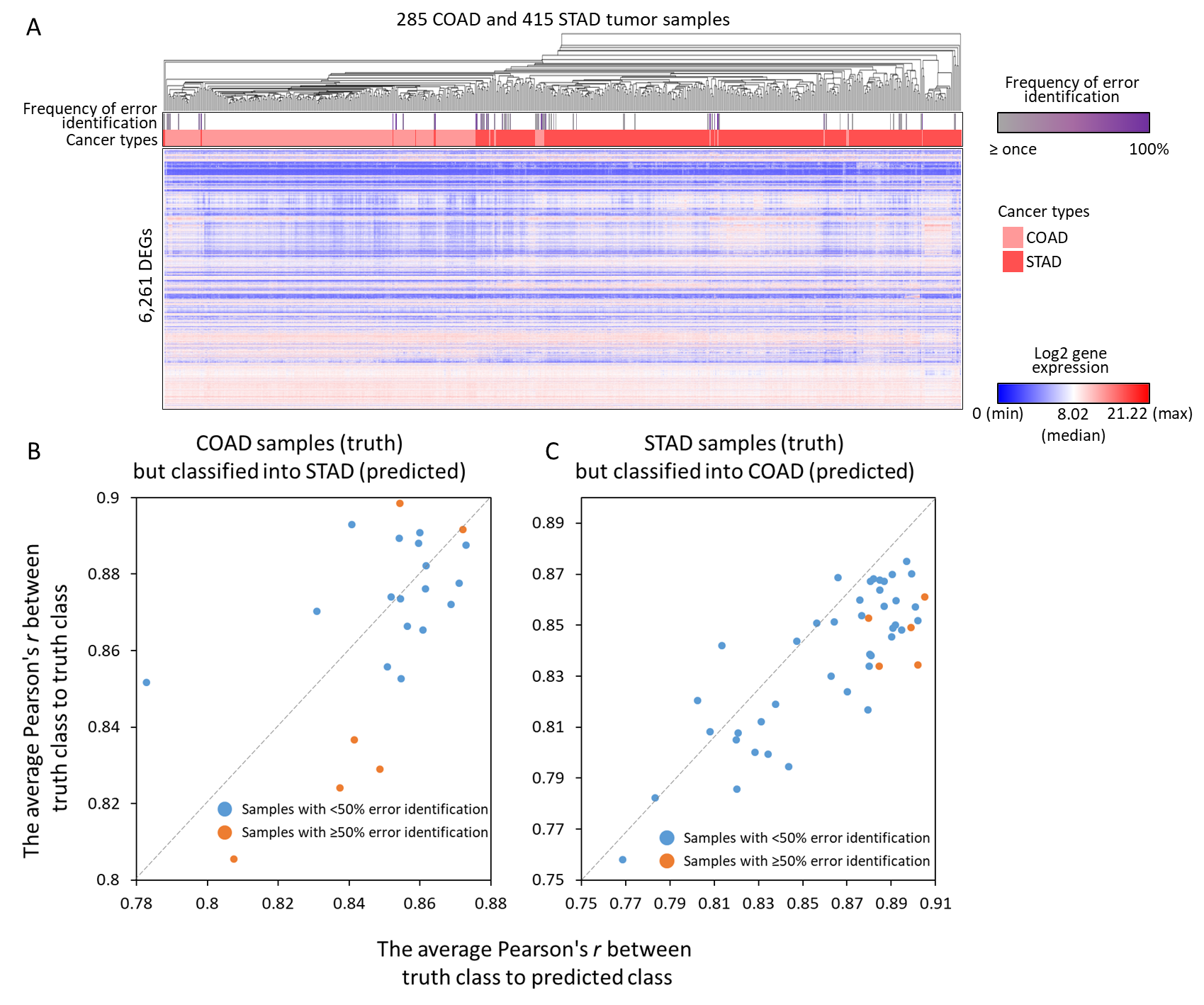


**Figure S4.** The correlation analysis of gene expression profiles between misjudged tumors and tumors in truth and predicted classes. (A) The heat map showed the unsupervised clustering analysis of gene expression in COAD and STAD tumor samples. The intensity of gene expression for 6,261 DEGs are presented in red color and blue color. To the top panels, two colors indicate COAD (pink color) and STAD (crimson color), respectively. And the frequency of error identification is presented in purple color. In the two scatter plots (B and C), each dot represents the each misjudged tumor sample, and the orange dots means their frequencies of error identification ≥ 50% among the number of total prediction times, otherwise the ones were presented with blue dots. The plots illustrated the averaged gene expression correlation (i.e., Pearson’s *r*) between the each misjudged tumor of (B) COAD and (C) STAD to every tumor in truth class cancers and in predicted class cancers. If the dots are below the diagonal that indicate the gene expression profiles of misjudged tumors are more similar to tumors in predicted classes than in truth classes.

**
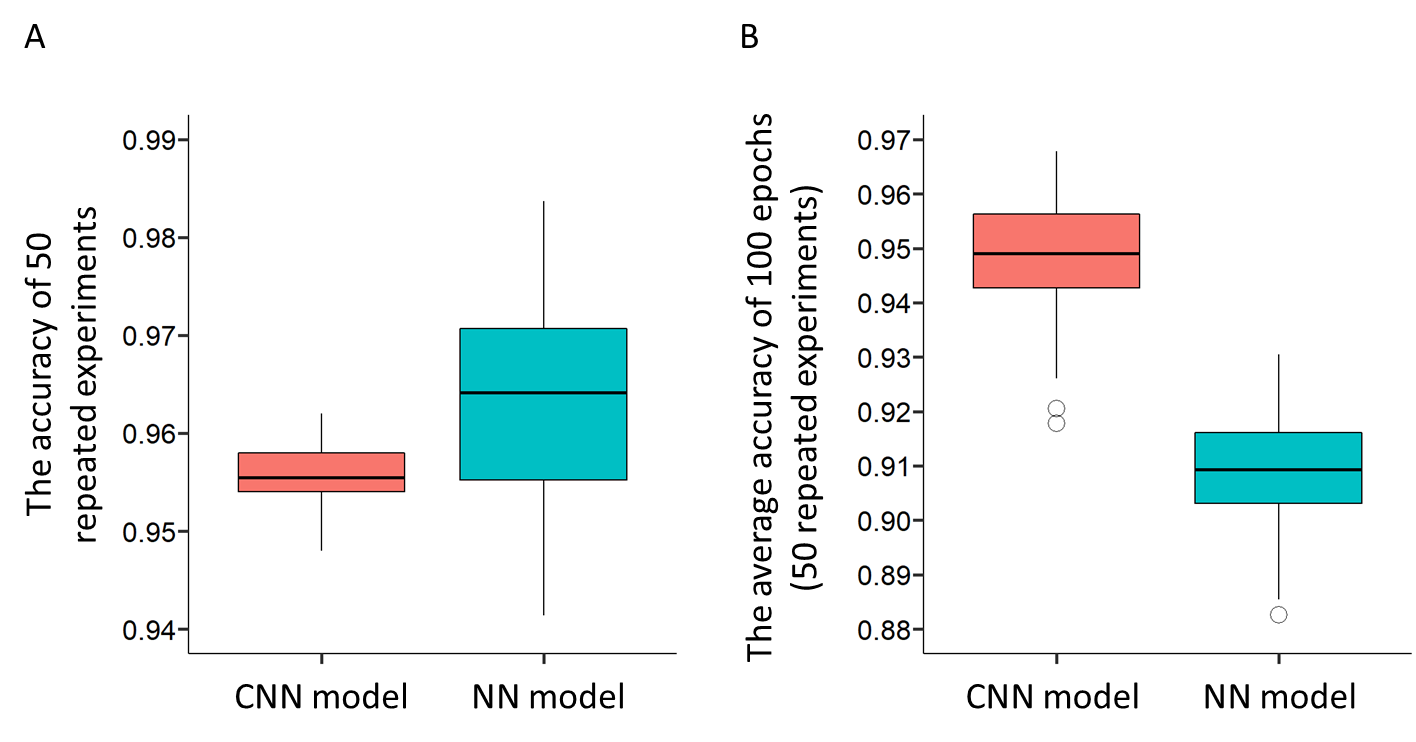
**

**Figure S5.** The accuracy values of 50 repeated experiments for CNN and NN model. (A) The best accuracy of 100 epochs was recorded in each individual repeated experiment. The variance and SD of 50 accuracy values were 1.11E-04 and 1.05E-02 for NN model, and the ones were 1.05E-05 and 3.25E-03 for CNN model. (B) The average accuracy of 100 epochs was recorded in each individual repeated experiment.

**Table S1.** The comparison of CNN model.

|  | Mostavi et al. (BMC Med Genomics . 2020 Apr 3;13(Suppl 5):44.)^1^ | Matsubara et al. (J Bioinform Comput Biol . 2019 Jun;17(3):1940007.)^2^ | Our study |
| --- | --- | --- | --- |
| CNN model | Three CNN model: (1) 1D-CNN; (2) 2D-Vanilla-CNN; (3) 2D-Hybrid-CNN | Model is similar to 2D-Vanilla-CNN of Mostavi et al., but has **two more convolution and pooling layers**. | Our model is similar to the ones of Matsubara et al. and we  modified CNN structures to predict multi-cancer types. |
| PPI network source | Authors **did not** use PPI data. | PPIs were collected from HINT 4.0 database (PMID: 22846459)^3^. | PPIs were collected from five public databases, BioGRID^4^, DIP^5^, IntAct^6^, MINT^7^ and MIPS^8^. |
| Input data | Gene expression profiles of **7,091 genes** were reformatted to (1) a vector with 1x7,100 cells; or (2) a matrix with 100x71 cells. | The PPI network **(3,042 nodes)** and gene expression profiles were mapped into 2D representation of 100x100 cells as model input. | The PPI network **(1,849 nodes)** and gene expression profiles, were mapped into 2D representation of 100x100 cells as model input. |
| Data pre-processing | Across all TCGA samples, the genes whose mean of FPKM <0.5 or standard deviation of FPKM <0.8, were removed. Finally, 7,091 genes were selected and reformatted to vector and matrix as model input. | Authors **did not** filter any genes. | We utilized \|fold change\| ≥2 and p-value <0.01 to identify differentially expressed genes (DEGs) between tumors and normal tissues for 11 cancer types. Finally, we combine these DEG genes and PPI networks. |
| Multi-label classification | 33 cancer types and normal tissue | Only two types (Lung cancer tumor vs normal tissue) | 11 cancer types and normal tissue |
| Prediction accuracy | Prediction accuracies = 93.9 to 95.0% among 34 classes (33 cancers and normal). | Prediction accuracy = 81% for 2 classes prediction (cancer samples vs non-cancer samples). | Prediction accuracies are 97.4% (normal samples vs. tumor samples) and 95.4% for 12 classes (11 cancer types and normal). |

**Reference**

1 Mostavi, M., Chiu, Y. C., Huang, Y. & Chen, Y. Convolutional neural network models for cancer type prediction based on gene expression. *BMC Med Genomics* **13**, 44, doi:10.1186/s12920-020-0677-2 (2020).

2 Matsubara, T., Ochiai, T., Hayashida, M., Akutsu, T. & Nacher, J. C. Convolutional neural network approach to lung cancer classification integrating protein interaction network and gene expression profiles. *J Bioinform Comput Biol* **17**, 1940007, doi:10.1142/S0219720019400079 (2019).

3 Das, J. & Yu, H. HINT: High-quality protein interactomes and their applications in understanding human disease. *BMC Syst Biol* **6**, 92, doi:10.1186/1752-0509-6-92 (2012).

4 Stark, C. *et al.* The BioGRID Interaction Database: 2011 update. *Nucleic Acids Res* **39**, D698-704, doi:10.1093/nar/gkq1116 (2011).

5 Xenarios, I. *et al.* DIP, the Database of Interacting Proteins: a research tool for studying cellular networks of protein interactions. *Nucleic Acids Res* **30**, 303-305, doi:10.1093/nar/30.1.303 (2002).

6 Aranda, B. *et al.* The IntAct molecular interaction database in 2010. *Nucleic Acids Res* **38**, D525-531, doi:10.1093/nar/gkp878 (2010).

7 Ceol, A. *et al.* MINT, the molecular interaction database: 2009 update. *Nucleic Acids Res* **38**, D532-539, doi:10.1093/nar/gkp983 (2010).

8 Mewes, H. W. *et al.* MIPS: analysis and annotation of genome information in 2007. *Nucleic Acids Res* **36**, D196-201, doi:10.1093/nar/gkm980 (2008).
