# Supplementary figures and images for: Convolutional neural network for human cancer types prediction by integrating protein interaction networks and omics data
Source: Sci Rep. 2021 Oct 19;11:20691. doi: 10.1038/s41598-021-98814-y (PMC8526703; doi:10.1038/s41598-021-98814-y)

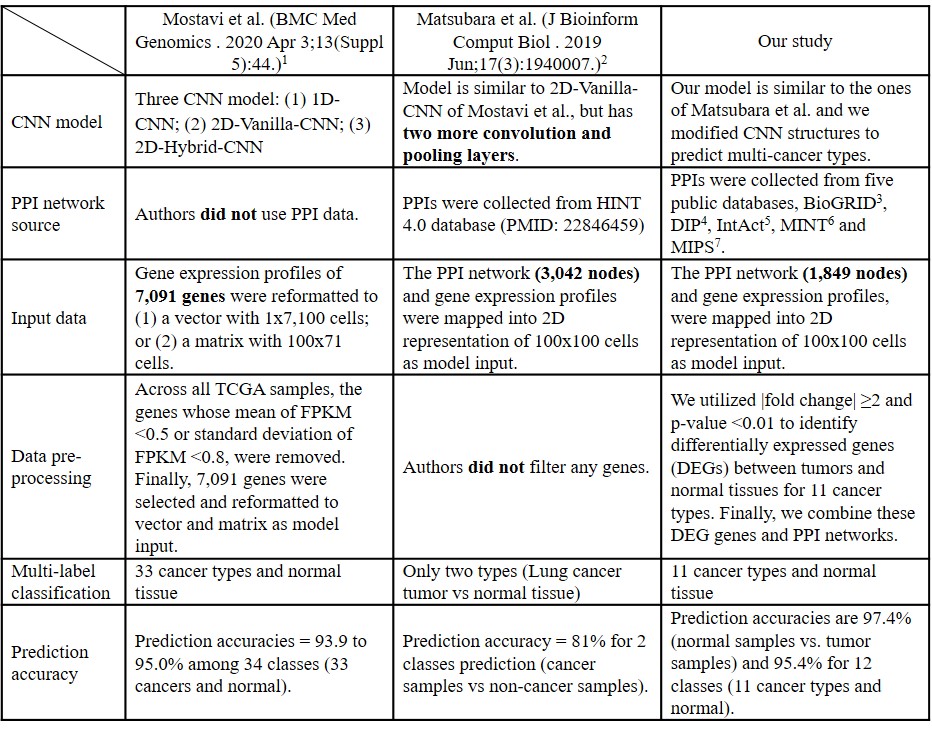

Supplement: Supplementary file 3 — Supplementary Information 3. [file 41598_2021_98814_MOESM3_ESM.jpg]
